# Auricular acupressure and VR for preventing preoperative constipation in Stanford type B aortic dissection

**DOI:** 10.1097/MD.0000000000043822

**Published:** 2025-08-08

**Authors:** Dan Shen, Kaiping Lu, Hao Wu, Chenying Xie, Yufen Wang, Lei Chen, Xuelin Wang, Haiyun Wang

**Affiliations:** a Department of Vascular Surgery, General Surgery, Cancer Center, Zhejiang Provincial People’s Hospital (Affiliated People’s Hospital), Hangzhou Medical College, Hangzhou, Zhejiang Province, China.

**Keywords:** auricular acupressure, nursing satisfaction, preoperative constipation, stanford type B aortic dissection, virtual reality technology

## Abstract

Preoperative constipation in patients with Stanford type B aortic dissection (TBAD) poses significant risks by increasing abdominal pressure, potentially complicating aortic stability. Traditional constipation management may be limited by both physiological and psychological factors. This study evaluates the efficacy of auricular acupressure combined with virtual reality (VR) technology in preventing preoperative constipation in TBAD patients, exploring its impact on both constipation severity and nursing satisfaction. This retrospective study included 116 patients with TBAD who received preoperative care at our hospital from January 2022 to December 2023. Participants were divided into 2 groups: a control group (n = 60) receiving standard care and an observation group (n = 56) receiving auricular acupressure combined with VR in addition to standard care. Data on constipation severity were collected pre- and posttreatment using the Wexner Constipation Score and Bristol Stool Form Scale, while nursing satisfaction was evaluated through a custom questionnaire. Statistical analyses were performed using SPSS Version 27.0 (Chicago). Pretreatment constipation scores showed no significant differences between groups. Posttreatment, however, the observation group demonstrated significantly improved scores for both the Wexner Constipation Score (from 12.94 ± 3.28 to 4.14 ± 1.23, *P* < .001) and the Bristol Stool Scale (from 1.50 ± 0.46 to 4.41 ± 1.39, *P* < .001) compared to the control group. Nursing satisfaction rates were also higher in the observation group, with an overall satisfaction rate of 94.6%, significantly surpassing the 78.3% in the control group (*P* = .010). Auricular acupressure combined with VR technology effectively reduces preoperative constipation severity and enhances nursing satisfaction in TBAD patients. This integrative, non-pharmacologic approach addresses both physiological and psychological factors, suggesting a valuable addition to conventional preoperative care protocols in this high-risk population.

## 1. Introduction

Stanford type B aortic dissection (TBAD) is a life-threatening vascular condition that occurs when a tear develops in the inner layer of the aorta’s wall, primarily affecting the descending thoracic aorta. This condition, typically distinguished from Type A dissections by the location of the tear, is often managed conservatively unless complications arise. Patients with TBAD, who often present with chronic hypertension and other cardiovascular risk factors, face a range of potential preoperative and postoperative complications.^[1–3]^ One common issue in these patients is preoperative constipation, a frequently underestimated complication that can significantly impact patient outcomes and comfort. The immobilization, stress, and dietary restrictions often associated with aortic dissection management contribute to constipation, which can lead to increased intra-abdominal pressure, thus affecting aortic stability and potentially worsening aortic dissection outcomes.^[4]^ Therefore, addressing preoperative constipation in this vulnerable group is essential for improving overall surgical outcomes and patient quality of life.

Traditional methods of constipation management, including dietary adjustments, laxatives, and physical activity, are often limited by the patient’s need for rest and restricted movement. Recent studies have explored non-pharmacological interventions to alleviate preoperative constipation, with auricular acupressure emerging as a promising approach. Auricular acupressure, rooted in traditional Chinese medicine (TCM), involves stimulating specific points on the ear believed to influence gastrointestinal motility, reduce stress, and improve autonomic nervous system balance.^[5]^ Its noninvasive nature and low risk of adverse effects make it an appealing alternative, particularly in patients with severe cardiovascular conditions like TBAD, where pharmacologic interventions must be carefully monitored to avoid aggravating hemodynamic instability.^[6,7]^ However, while auricular acupressure has shown benefits in enhancing gastrointestinal motility, its effects on preventing preoperative constipation in patients with TBAD remain inadequately studied. In recent years, virtual reality (VR) technology has emerged as an innovative approach in healthcare, widely recognized for its capacity to reduce anxiety, alleviate pain, and promote relaxation. VR engages patients in immersive environments, providing psychological and physiological benefits that can positively influence gastrointestinal function. Through mechanisms of distraction and relaxation, VR helps lower sympathetic nervous system activity, which in turn may reduce stress-induced motility inhibition. In preoperative settings, VR has shown promise in managing anxiety and facilitating pain relief, suggesting its potential utility in enhancing gastrointestinal outcomes, particularly in high-stress patient groups like those with TBAD.^[8,9]^

Combining auricular acupressure with VR technology could offer a synergistic approach to managing preoperative constipation by simultaneously addressing both physiological and psychological factors that contribute to gastrointestinal dysmotility. The physiological effects of auricular acupressure on gastrointestinal motility may be enhanced by the relaxation and stress-relieving effects of VR, potentially providing a comprehensive intervention for constipation prevention in TBAD patients. Moreover, a combined approach may offer an alternative to traditional pharmacological treatments, which carry risks in this delicate patient population. This study aims to evaluate the efficacy of auricular acupressure combined with VR technology in preventing preoperative constipation among patients with Stanford type B aortic dissection.

## 2. Methods

### 2.1. Study design

This study was a retrospective observational study, approved by the Ethics Committee of Zhejiang Provincial People’s Hospital. A retrospective evaluation was conducted at our hospital to assess the efficacy of auricular acupressure combined with VR technology in the prevention of preoperative constipation among patients with TBAD. This study analyzed data collected over a 24-month period, from January 2022 to December 2023. A total of 116 patients diagnosed with Stanford type B aortic dissection met the inclusion criteria and were enrolled in the study. Participants were divided into 2 groups based on the preoperative interventions they had voluntarily received in routine clinical practice: the control group (n = 60), which received standard care, and the observation group (n = 56), which received standard care plus auricular acupressure combined with VR technology. The study’s design, objectives, and protocols were established following the STROBE (Strengthening the Reporting of Observational Studies in Epidemiology) guidelines to ensure methodological rigor and transparency. The research plan received ethical approval from the Ethics Committee of our hospital, confirming adherence to ethical standards and patient safety considerations.^[10]^

### 2.2. Inclusion and exclusion criteria

Inclusion criteria:

Patients diagnosed with Stanford type B aortic dissection (TBAD) confirmed by thoracic aorta computed tomography (CT) or CT angiography (CTA).

Diagnosis of chronic constipation, defined by meeting at least 2 of the following criteria: straining during at least 25% of defecations; passage of lumpy or hard stools in at least 25% of defecations; sensation of incomplete evacuation in at least 25% of defecations; sensation of anorectal blockage or colonic obstruction in at least 25% of defecations. Fewer than 3 defecations per week on average.

Age ≥ 18 years, with no gender restrictions.

Patients demonstrating a willingness to actively cooperate with clinical treatment and nursing care.

Exclusion criteria:

History of organic lesions in the rectum or colon.

Constipation caused by mesenteric ischemia, other acute abdominal conditions, or organic gastrointestinal diseases.

Severe dysfunction of critical organs, including the heart, liver, or kidneys.

Presence of consciousness disorders, mental illnesses, cognitive impairment, or advanced cancer.

Allergic constitution, inflammation, or skin damage in the auricle area preventing auricular acupressure application.

Inflammation, scarring, or other pathological changes in the external ear.

### 2.3. Intervention protocols

In the control group, patients received standard nursing care, which included dietary guidance, lifestyle adjustments, and medication to prevent constipation. Specifically, patients were encouraged to maintain adequate hydration, with a daily fluid intake of 1500 to 2000 mL, and to consume a diet low in fat, salt, and rich in fiber and water-soluble vitamins. Foods high in fat, spices, raw, or cold items were discouraged. For those with normal blood glucose levels, honey water or warm saline was recommended each morning to facilitate bowel movements. Patients were also advised to establish a regular bowel schedule and maintain a semi-sitting or lying position during defecation to avoid prolonged straining, as well as to remain emotionally calm, abstain from smoking and alcohol, and prevent infections. Auricular acupressure was also employed, targeting specific points such as the rectum, colon, and sympathetic points, as outlined in the “Chinese Pharmacopoeia” (GB/T13734-92). Acupressure beans were applied on these points with adhesive tape and massaged 5 times daily, with beans being replaced based on seasonal timing.

In addition to standard care, patients in the observation group received auricular acupressure combined with VR technology to further aid in the prevention of preoperative constipation. An integrated management team, including a senior physician and 5 experienced nurses, was formed to oversee training and care in aortic dissection management, auricular acupressure, and VR application. The “Aidu APP,” a cross-platform VR-based auricular acupressure training system, was developed to guide patients through modules such as “Virtual Simulation Training,” “VR-guided relaxation module,” and “Database.” High-quality 360-degree VR videos provided a patient’s perspective on acupressure techniques, detailing points, steps, and best practices. Through the VR training module, patients engaged in interactive training sessions with real-time feedback, including visual and auditory cues to reinforce proper technique, and background music to enhance comfort. The VR-guided relaxation module created a relaxed bathroom-like environment with soft lighting and calming music to reduce anxiety and promote comfort during bowel movements. Additionally, the database module enabled patients to log their adherence, tracked accuracy in acupressure application, and provided real-time physiological and psychological feedback to support individualized care adjustments based on treatment progress.

### 2.4. Data collection and outcome measures

Data collection included a comparative assessment of constipation in both groups before and after treatment. Constipation severity was assessed using the Wexner Constipation Scoring System and the Bristol Stool Form Scale.^[11,12]^ The Wexner system evaluates symptoms such as bowel movement frequency, evacuation difficulty, pain during defecation, and need for assistance, with scores ranging from 0 (no constipation) to 30 (severe constipation), where higher scores indicate greater severity. The Bristol Stool Form Scale categorizes stool types from Type 1 (severe constipation) to Type 7 (severe diarrhea), with Types 3 to 4 representing normal consistency. Together, these tools provide a comprehensive evaluation of bowel habits and stool consistency, allowing for a clear assessment of constipation severity and the effects of treatment interventions. Patient satisfaction with nursing services was also measured through a custom-designed questionnaire with a total score of 100 points, categorized as follows: scores of 90 to 100 indicated very satisfied, 60 to 89 indicated satisfied, and 0 to 59 indicated dissatisfied.

### 2.5. Statistical analysis

Statistical analyses were conducted with SPSS software (Version 27.0) to evaluate study data. Initially, data were categorized as either quantitative or categorical, and normality tests were employed to determine distribution patterns. For quantitative data following a normal distribution, inter-group comparisons were made using independent sample t-tests, with results reported as mean ± standard deviation. Categorical data were presented as frequencies and percentages, and relationships or independence among these variables were assessed using Chi-square (χ²) tests; if Chi-square test assumptions were not met, Fisher exact test was applied. All statistical tests were 2-tailed, and significance was determined at a *P*-value of <.05.

## 3. Results

### 3.1. Clinical characteristics of control and observation groups

In this study, the clinical characteristics of patients in the control and observation groups were assessed and compared to ensure baseline comparability. Notably, the classification of AD complexity showed a similar distribution in both groups, with a higher percentage of noncomplex cases (55.0% in the control group and 60.7% in the observation group) compared to high-risk and complex cases. This distribution supports comparable initial risk stratification across groups. Demographically, both groups were similar in age, with mean ages of 57.3 ± 10.2 years in the control group and 56.8 ± 9.8 years in the observation group. Gender distribution also showed consistency, with males constituting the majority in each group (66.7% in the control group and 67.9% in the observation group). Body mass index was similarly distributed, with medians indicating a stable and comparable baseline for both groups. The prevalence of common comorbidities, including hypertension, diabetes, hyperlipidemia, coronary artery disease, and stroke, was similar between groups, with hypertension being the most common (86.7% in the control group and 82.1% in the observation group). Other underlying health conditions, such as liver dysfunction, renal dysfunction, and histories of smoking and alcohol use, showed no significant disparities between groups, suggesting minimal bias related to preexisting health risks. Additionally, the incidence of liver and renal tumors, as well as pleural effusion and ascites, was low and comparably distributed, with both groups presenting similar rates (Table 1). Overall, the control and observation groups were well-matched in terms of demographic, clinical, and comorbid characteristics, providing a solid foundation for subsequent analysis and interpretation of intervention outcomes.

**Table 1:** Clinical characteristics of control and observation groups.

Item	Control group (n = 60)	Observation group (n = 56)
AD classification		
Complex	4 (6.7)	2 (3.6)
High risk	23 (38.3)	20 (35.7)
Noncomplex	33 (55.0)	34 (60.7)
Age (year)	57.3 ± 10.2	56.8 ± 9.8
Male	40 (66.7)	38 (67.9)
BMI (kg/m²)	22.4 (21.2, 24.5)	22.7 (20.6, 24.4)
Hypertension	52 (86.7)	46 (82.1)
Diabetes	4 (6.7)	3 (5.4)
Hyperlipidemia	2 (3.3)	3 (5.4)
CAD	13 (21.7)	10 (17.9)
Stroke	11 (18.3)	8 (14.3)
Liver dysfunction	4 (6.7)	2 (3.6)
Renal dysfunction	3 (5.0)	2 (3.6)
Smoking history	30 (50.0)	24 (42.9)
Alcohol history	29 (48.3)	20 (35.7)
Liver tumor	2 (3.3)	3 (5.4)
Renal tumor	7 (11.7)	5 (8.9)
Pleural effusion	18 (30.0)	12 (21.4)
Ascites	3 (5.0)	2 (3.6)

BMI = body mass index, CAD = coronary artery disease.

### 3.2. Comparison of constipation scores before and after treatment between control and observation groups

Analysis of constipation severity, assessed through the Wexner Constipation Score and the Bristol Stool Scale Score, showed significant differences in outcomes between the control and observation groups following treatment. Initially, there were no statistically significant differences in pretreatment scores for either the Wexner Constipation Score (*t* = 0.543, *P* = .588) or the Bristol Stool Scale Score (*t* = 0.957, *P* = .340), indicating comparable baseline constipation severity between groups. Posttreatment analysis, however, revealed substantial improvements in both measures within each group, with the observation group achieving significantly better results. For the Wexner Constipation Score, the control group showed a reduction from 13.24 ± 2.65 to 8.71 ± 2.41 (*P* < .05), while the observation group decreased more markedly from 12.94 ± 3.28 to 4.14 ± 1.23, with an inter-group difference that was statistically significant (*t* = 12.73, *P* < .001). Similarly, the Bristol Stool Scale Score improved in both groups posttreatment, rising from 1.42 ± 0.44 to 2.61 ± 0.83 in the control group (*P* < .05) and from 1.50 ± 0.46 to 4.41 ± 1.39 in the observation group. The posttreatment difference between groups was also statistically significant (*t* = 8.534, *P* < .001) (Table 2). These findings suggest that the intervention administered to the observation group was highly effective in reducing constipation severity and improving stool consistency, with both scores indicating a more pronounced improvement compared to the control group. This outcome underscores the potential efficacy of the combined intervention in managing preoperative constipation.

**Table 2:** Comparison of Wexner constipation score and Bristol stool scale score before and after treatment between control and observation groups (x̄ ± s).

Measure	Control group (n = 60)	Observation group (n = 56)	*t*-value	*P*-value
Wexner constipation score				
Before treatment	13.24 ± 2.65	12.94 ± 3.28	0.543	.588
After treatment	8.71 ± 2.41*	4.14 ± 1.23*	12.73	<.001
Bristol stool scale score				
Before treatment	1.42 ± 0.44	1.50 ± 0.46	0.957	.340
After treatment	2.61 ± 0.83*	4.41 ± 1.39*	8.534	<.001

* Compared with pretreatment values within the same group, *P* < .05.

### 3.3. Comparison of nursing satisfaction between control and observation groups

The comparison of nursing satisfaction between the control and observation groups demonstrated a significantly higher satisfaction rate in the observation group following intervention. The overall satisfaction rate (defined as the combined percentages of “Satisfied” and “Highly Satisfied” responses) reached 94.6% in the observation group, substantially higher than the 78.3% observed in the control group. This difference was statistically significant (χ² = 6.480, *P* = .010). In terms of specific satisfaction levels, the observation group had a notably lower proportion of “Dissatisfied” responses (3 vs 13) compared to the control group. Additionally, the number of participants reporting “Highly Satisfied” responses was more than double in the observation group compared to the control group (18 vs 8) (Table 3). These results suggest that the intervention was effective in enhancing patient satisfaction with nursing care, potentially attributable to the comprehensive and supportive approach incorporated in the observation group’s treatment protocol.

**Table 3:** Comparison of nursing satisfaction between the study and control groups.

Group	Dissatisfied (n)	Basic satisfaction (n)	Satisfied (n)	Highly satisfied (n)	Satisfaction rate (%)
Control group (n = 60)	13	21	18	8	78.3
Observation group (n = 56)	3	13	22	18	94.6
χ² value	–	–	–	–	6.480
*P*-value	–	–	–	–	.010

## 4. Discussion

Preoperative constipation is a common yet often overlooked issue in patients preparing for surgery, particularly in those with TBAD. Constipation in these patients not only causes discomfort but may also increase abdominal pressure, potentially compromising the stability of the dissection site and heightening the risk of complications. Traditional management of preoperative constipation often relies on dietary modifications and pharmacologic interventions, which, while effective, may not fully address the psychosomatic contributors to constipation, such as stress and anxiety, that are prevalent in the preoperative period.^[13,14]^ Auricular acupressure, a noninvasive therapy rooted in TCM, has demonstrated promising effects on gastrointestinal motility by stimulating reflex points on the ear associated with digestive function. This technique aligns well with the needs of TBAD patients, who may benefit from non-pharmacologic therapies to mitigate constipation without impacting hemodynamic stability.^[15,16]^ Additionally, the incorporation of VR technology presents an innovative approach, allowing patients to experience immersive, calming environments that can alleviate preoperative stress. The findings of this study suggest that auricular acupressure combined with VR technology can significantly improve constipation symptoms and patient satisfaction in those with TBAD undergoing preoperative care.

Auricular acupressure has roots in TCM and is recognized for its regulatory effects on gastrointestinal motility. It works by stimulating specific acupressure points on the ear that are believed to correspond to various bodily functions, including bowel movement. The reflexive stimulation of points associated with the rectum, colon, and sympathetic nervous system may help restore normal peristalsis by activating the vagal nerve pathway. This parasympathetic activation may counteract stress-induced sympathetic dominance, which is known to inhibit gastrointestinal function. Through this mechanism, auricular acupressure could help alleviate chronic constipation by promoting bowel motility in patients who otherwise experience delayed gastric transit. The addition of VR technology in this context further amplifies the therapeutic effect. VR provides an immersive environment that can help reduce stress and anxiety by promoting a state of relaxation. Given that stress is a known contributor to constipation, particularly in preoperative patients who may be anxious about their upcoming surgery, VR’s capacity to create a calming, engaging experience may reduce the sympathetic activation associated with stress.^[17,18]^ By reducing stress levels, VR indirectly supports improved gastrointestinal motility and function, helping patients achieve better stool consistency and more regular bowel movements. The significant improvements observed in the observation group’s Bristol Stool Scale scores suggest that this dual-intervention approach effectively addressed both the physiological and psychological contributors to constipation in TBAD patients.

The enhanced nursing satisfaction reported in the observation group suggests that patients felt more supported and cared for within this intervention framework. This improvement in satisfaction is likely attributable to the multifaceted nature of the intervention, which not only addressed constipation symptoms but also promoted a holistic, patient-centered approach. By combining auricular acupressure, a familiar therapeutic technique, with innovative VR technology, the intervention may have met both the physical and emotional needs of patients, fostering a positive perception of care. VR’s immersive training modules and simulated defecation scenarios might have enhanced patients’ sense of autonomy and control over their symptoms, thus improving satisfaction with the nursing care provided.^[19,20]^ The high satisfaction levels in the observation group could have further implications for clinical outcomes. Increased satisfaction is often linked to higher adherence to care protocols, which is crucial in managing conditions like TBAD, where optimal surgical outcomes depend on stringent preoperative preparation.^[21,22]^ Patients who feel satisfied with their care are more likely to adhere to prescribed interventions and engage actively in preoperative procedures, potentially leading to fewer complications, a smoother recovery, and overall improved prognosis. This highlights the broader impact of patient-centered, satisfaction-focused interventions in surgical preparation.

While the results indicate promising benefits, there are several limitations to consider. First, this study’s retrospective design may introduce selection bias, confounding variables, and limits the ability to establish a definitive causal relationship between the intervention and the outcomes. Second, patients were not randomly assigned to groups, and the choice of intervention was based on their prior voluntary decisions, which may further contribute to bias. Additionally, the sample size was relatively small and limited to a single center, which restricts the generalizability of the findings to broader surgical populations or patients with other types of aortic dissection. Therefore, we strongly recommend that future research be conducted through large-scale, prospective, randomized controlled trials to verify the causal relationship between auricular acupressure, VR interventions, and improvements in preoperative constipation. Furthermore, randomized controlled trials would help to isolate the individual effects of each intervention component and validate the replicability and efficacy of this combined approach across diverse clinical settings.

## 5. Conclusions

The combination of auricular acupressure with VR technology demonstrates significant efficacy in reducing preoperative constipation and enhancing nursing satisfaction in patients with Stanford type B Aortic Dissection. This synergistic, non-pharmacologic approach addresses both physiological and psychological factors, offering a valuable adjunct to conventional preoperative care and potentially improving surgical outcomes in this high-risk population.

## Author contributions

**Conceptualization:** Dan Shen, Kaiping Lu, Hao Wu, Lei Chen, Haiyun Wang.

**Data curation:** Dan Shen, Hao Wu, Lei Chen.

**Formal analysis:** Dan Shen, Kaiping Lu, Hao Wu, Yufen Wang, Haiyun Wang.

**Funding acquisition:** Dan Shen, Kaiping Lu.

**Investigation:** Dan Shen, Kaiping Lu, Chenying Xie, Yufen Wang, Haiyun Wang.

**Methodology:** Dan Shen, Chenying Xie, Xuelin Wang.

**Supervision:** Yufen Wang.

**Validation:** Kaiping Lu, Xuelin Wang.

**Visualization:** Lei Chen.

**Writing – original draft:** Dan Shen, Kaiping Lu.

**Writing – review & editing:** Dan Shen, Kaiping Lu.
